# Do maternal anxiety and depressive symptoms predict anxiety in children with and without ADHD at 8 years?

**DOI:** 10.1007/s00787-024-02374-1

**Published:** 2024-02-20

**Authors:** Christine Baalsrud Ingeborgrud, Beate Oerbeck, Svein Friis, Are Hugo Pripp, Pål Zeiner, Heidi Aase, Guido Biele, Søren Dalsgaard, Kristin Romvig Overgaard

**Affiliations:** 1 Institute of Clinical Medicine, University of Oslo, Oslo, Norway; 2 Division of Mental Health and Addiction, Oslo University Hospital, Oslo, Norway; 3 Oslo Centre of Biostatistics and Epidemiology, Oslo University Hospital, Oslo, Norway; 4 Department of Child Health and Development, Norwegian Institute of Public Health, Oslo, Norway; 5 Institute of Clinical Medicine, University of Copenhagen, Copenhagen, Denmark; 6 Dept. of Child and Adolescent Psychiatry Glostrup, Mental Health Services of the Capital Region, Hellerup, Denmark; 7 School of Business and Social Sciences, National Centre for Register-Based Research, Aarhus University, Aarhus C, Denmark; 8 Division of Mental Health and Addiction, Child and Adolescent Psychiatry Unit, Institute of Clinical Medicine, University of Oslo, Blindern, 0315 Oslo, Norway

**Keywords:** Child anxiety, Maternal anxiety and depression, Attention-deficit, Hyperactivity disorder

## Abstract

Maternal anxiety and depression during pregnancy and early childhood have been associated with child anxiety and attention-deficit/hyperactivity disorder (ADHD). However, previous studies are limited by their short follow-up, few assessments of maternal symptoms, and by not including maternal and child ADHD. The present study aimed to fill these gaps by investigating whether maternal anxiety and depressive symptoms from pregnancy to child age 5 years increase the risk of child anxiety disorders at age 8 years. This study is part of the population-based Norwegian Mother, Father, and Child Cohort Study. Maternal anxiety and depressive symptoms were assessed by the Hopkins Symptom Checklist (SCL) six times from pregnancy through early childhood, and ADHD symptoms by the Adult Self-Report Scale (ASRS). At age 8 years (*n* = 781), symptoms of anxiety disorders and ADHD were assessed, and disorders classified by the Child Symptom Inventory-4. Logistic regression models estimated the risk of child anxiety depending on maternal symptoms. The mothers of children classified with an anxiety disorder (*n* = 91) scored significantly higher on the SCL (at all time points) and ASRS compared with the other mothers. In univariable analyses, maternal anxiety and/or depression and ADHD were associated with increased risk of child anxiety (odds ratios = 2.99 and 3.64, respectively), remaining significant in the multivariable analysis adjusted for covariates. Our findings link maternal anxiety, depression, and ADHD during pregnancy and early childhood to child anxiety at age 8 years.

**Supplementary Information:**

The online version contains supplementary material available at 10.1007/s00787-024-02374-1.

## Introduction

Maternal anxiety and depression during pregnancy and early childhood are known to negatively impact offspring [1, 2]. During the perinatal period, maternal anxiety and depression are common disorders, and are estimated to occur in 20–25% of mothers [3, 4]. In high-income countries, approximately 13% of mothers experience perinatal clinical anxiety and 5% experience depression [5]. During the child age intervals from 2 to  < 4, 4 to  < 6, and 6 to  < 8 years, a nationally representative British study found the prevalence of maternal anxiety and depression to be approximately 8% and 18%, respectively [6].

One interest in the perinatal period originates from the assumption that maternal symptoms may expose the fetus to a risk of psychiatric disorders in childhood through a diverse range of biological mechanisms. Indeed, an abundance of literature links maternal perinatal anxiety and depression to child internalizing symptoms (such as anxiety and depression), with a small-to-medium effect size reported in a recent meta-analysis [7].

However, Rogers et al. highlighted that previous studies primarily focused on maternal symptom associations with offspring symptoms during early childhood [7]; there is, therefore, a need for future studies to explore symptoms in offspring at a later age. A study that followed child internalizing symptoms from age 2 to 11 years (*n* = 1,364) found that maternal postpartum anxiety and depressive symptoms only modestly differentiated between the child internalizing pathways (low-stable, increasing/decreasing, and high-stable trajectories) [8]. However, that study only examined maternal symptoms from one time point (one month after birth) and stressed that further data were needed. Nevertheless, the importance of maternal symptoms for child mental health after the perinatal period was highlighted in a study of Canadian children (*n* = 937), in which offspring exposed to maternal depression between the ages of 2–3 and 4–5 years had a twofold increase in odds of emotional disorder at age 12–13 years [9]. Similarly, an Australian study that interviewed mothers (*n* = 4,434) repeatedly during gestation up to child age 14 years found that maternal anxiety and depression during early childhood were associated with a significant risk for the development of anxiety and depression symptoms in their offspring, and even more so with repeated exposure to higher maternal symptoms [1].

High co-occurrence rates have been found between anxiety, depression, and attention-deficit/hyperactivity disorder (ADHD) in adults generally [10, 11], and also specifically in mothers [12]. Not surprisingly, several studies have documented an association between maternal anxiety and depression and child ADHD [13–15]. However, despite these known associations, to our knowledge, analyses combining measures of maternal ADHD with those of anxiety and depression in the study of child anxiety disorders are lacking. Furthermore, as noted by Rogers et al. [7], few studies have examined the outcome of internalizing disorders in middle childhood and, to our knowledge, none has used diagnostic criteria for child anxiety disorders. Previous studies were also limited by the number of time points (mostly one or two) at which maternal symptoms were reported and were thus unable to address whether transient or repeated episodes of anxiety and depression are associated with the highest risk for offspring anxiety.

The present study aimed to fill these knowledge gaps by investigating whether maternal anxiety and depressive symptoms from pregnancy to child age 5 years increase the risk of child anxiety disorders at age 8 years. We first hypothesized that there would be such an increased risk, and second that the risk would be stronger depending on (i) maternal symptoms of ADHD and (ii) child ADHD. Finally, we hypothesized that repeated episodes of maternal anxiety and/or depression were associated with an increased risk for child anxiety disorders. To avoid an overestimation of associations, we included both current maternal symptoms and child symptoms of anxiety and ADHD at 3 years in the analyses.

## Methods

### Participants

The Norwegian Mother, Father, and Child Cohort Study (MoBa) [16] is a prospective population-based cohort study of Norwegian-speaking pregnant women conducted by the Norwegian Institute of Public Health, which started in 1999 (41% participation rate of pregnant women invited during the 10-year recruitment period) [17]. The ADHD Study, a sub-study from MoBa, was described previously [18, 19]. In the sub-study, mothers of children scoring high on ADHD symptoms on the 3-year-MoBa questionnaire were invited, along with mothers of children selected randomly from MoBa. High-scorers (‘screen-positive’ children) had ADHD symptoms scores ≥ 90th percentile on an 11-item screening measure for ADHD, which included six items from the Child Behavior Checklist/1.5–5 [20] and five items from the DSM-IV-TR criteria for ADHD [21] (*n* = 2,798), and were eligible for participation along with randomly selected children (*n* = 654). About 35% (*n* = 1,195) of the invited families participated in the initial on-site clinical assessment from 2007 to 2011, including diagnostic interviews with parents using the Preschool Age Psychiatric Assessment (PAPA) [22]. A small number of parents withdrew their consent to participate during the study, leaving 1,180 participants at the 8-year follow-up. In the current study, we combined information about the children from a diagnostic questionnaire in the ADHD sub-study when the children were 8 years old (responders were mainly mothers), with information from MoBa regarding mothers’ self-reports of symptoms of anxiety and depression at six time points from pregnancy until the child was aged 8 years. Included in this study were 781 children (66% of the participants at age 3 years) participating in the assessment at 8 years for whom there was available information about mothers’ anxiety and depression symptoms on at least one occasion.

## Measures

### Predictors

*Maternal anxiety and depression.* Maternal symptoms of anxiety and depression were assessed using short versions of the Hopkins Symptom Checklist, SCL-25 [23]. Mothers reported these symptoms on the eight-item version (SCL-8) at week 30 during pregnancy, and at child age 6 months, 18 months, 3 years, 5 years, and 8 years. At week 15 during pregnancy, mothers reported these symptoms on the five-item version (SCL-5). The SCL-8 consists of four items measuring depressive symptoms (‘Feeling hopeless about the future’, ‘Feeling blue’, ‘Worrying too much about things’, ‘Feeling everything is an effort’) and four measuring anxiety symptoms (‘Feeling fearful’, ‘Nervousness or shakiness inside’, ‘Feeling tense or keyed up’, ‘Suddenly scared for no reason’), whereas the SCL-5 comprises the first three depressive and the first two anxiety symptoms. Each item is rated on a four-point Likert scale (1–4) as occurring as ‘not at all’, ‘a little’, ‘quite a bit’, or ‘extremely’, leading to a range of scores of 5–20 for the SCL-5 and 8–32 for the SCL-8. The average item score was calculated by dividing the total score of the number of items answered, and the cut-off was set to ≥ 2 as consistent with suggestions for the SCL-5 [24]. The correlation between the SCL-8 and SCL-25 was reported to be as high as 0.94 [25], and the SCL-5 was found to perform almost equally well as the full version [24]. In our present sample, Cronbach’s alpha values ranged from 0.79 to 0.88 for the SCL-5 and SCL-8.

*Maternal ADHD symptoms.* Maternal symptoms of ADHD were measured by the six-item Adult Self-Report Scale (ASRS-6) at child age 3 years. The ASRS-6 comprises two factors, with four items measuring inattention and two items measuring hyperactivity–impulsivity (HI), consistent with the DSM-IV criteria [26]. The two-factor solution was confirmed in MoBa [27]. The items are scored on a five-point Likert scale (0–4) as occurring as ‘never’, ‘rarely’, ‘sometimes’, ‘often’, or ‘very often’, leading to a range of scores from 0 to 24. The ASRS-6 has a clinical cut-off of ≥ 14 [28]. Cronbach’s alpha in the current study was 0.70.

### Covariates

*Socio-economic factors.* Child birth date and sex were obtained from the Norwegian Medical Birth Registry. Length of parental education was obtained from the first MoBa assessment during pregnancy and converted to whether the mean length of the mother’s and father’s education was 12 years or more or less than 12 years (corresponding to whether parents have finished secondary school in Norway or not). Cohabitation status was obtained from the MoBa assessment at 8 years and reported as living with the father or not living with the father (as mainly reported by the mother).

*Preschool child symptoms.* At child age 3.5 years, parents were interviewed using the PAPA, and symptoms of anxiety and ADHD were scored as present or not present by trained psychology students. Symptoms were counted during the previous 3 months and then computed into a symptom sum score. In line with the PAPA guidelines, the anxiety sum score included seven symptoms of specific phobia, three symptoms of social phobia, seven symptoms of separation anxiety, and six symptoms of generalized anxiety. Following the algorithm for generalized anxiety, these symptoms were only counted when the criterion ‘worries’ was reported. Consistent with previous studies [19, 29], we used information from the PAPA and defined ADHD by the DSM-IV-TR criteria. [21]. We report the number of ADHD symptoms and classify the ADHD presentations by the presence of at least six out of nine symptoms of HI or inattention (IA). All correlations between predictors were low to moderate (Pearson *r* range, –0.09 to 0.33).

### Outcome measures at age 8 years

The parents completed the Child Symptom Inventory-4 (CSI-4), a questionnaire with items and algorithms for disorders derived from the DSM-IV diagnostic criteria [30]. The CSI-4 has been found to give reliable and valid measurements, with temporal stability over 4 years for most symptom categories [31]. The CSI-4 was rated on a four-point Likert scale (0–3) as occurring as ‘never’, ‘sometimes’, ‘often’, or ‘very often’ [30], and was dichotomized to being present or not present, in line with the algorithms in the CSI manual. The diagnostic cut-off scores were set to the minimum number of symptoms necessary for DSM-IV diagnoses of anxiety disorders and ADHD.

**Fig. 1:**
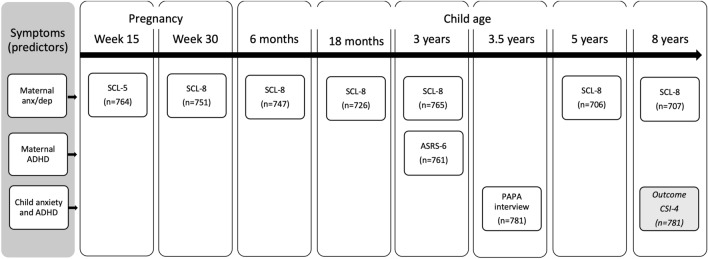
Timepoints for the measurements of predictors/maternal and child symptoms *SCL* short versions of the Hopkins Symptom Checklist, *ASRS-6* the Adult Self-Report Scale-6 for attention-deficit/hyperactivity disorder (ADHD), *PAPA interview* Preschool Age Psychiatric Assessment, *SCI-4* Child Symptom Inventory-4

*Outcome.* To be categorized with an anxiety disorder, the participants had to meet the criteria for at least one of the following: specific phobia, social phobia, separation anxiety, or generalized anxiety disorder. For specific phobia, the CSI-4 contains one symptom, which had to be present at least ‘sometimes’, according to the CSI-4 manual. In our sample, specific phobia was present ‘sometimes’ in 29.5% of the children, and we, therefore, required the symptom to be present ‘often’ or ‘very often’ for a participant to be categorized with specific phobia. For social phobia, the CSI-4 lists four symptoms, at least three of which had to be endorsed to fulfil the diagnostic criteria. For separation anxiety, the CSI-4 lists eight symptoms, at least three of which had to be endorsed. For generalized anxiety disorder, eight items are listed, where the modified criteria ‘Is over-concerned about abilities in academic, athletic or social activities’ or ‘Has difficulty controlling worries’ had to be endorsed for the other symptoms to be counted. For generalized anxiety disorder to be present, at least three symptoms had to be endorsed. For ADHD, the CSI-4 lists nine symptoms of IA and nine symptoms of HI, and at least six symptoms of IA or HI had to be endorsed. The study design with predictors and outcomes are presented in Fig. 1.

### Ethics

MoBa and the initial data collection were based on a license from the Norwegian Data Protection Agency and approval from the Regional Committees for Medical and Health Research Ethics. The MoBa cohort is currently regulated by the Norwegian Health Registry Act. The present study was approved by the Regional Committees for Medical and Health Research Ethics (2017/1276).

### Statistics

Pearson chi-square tests were used to compare categorical variables and *t* tests were used to compare means. First, univariable logistic regression was used in unadjusted models to estimate the odds of child anxiety at 8 years in the presence of the different predictors. Second, in the multivariable logistic regression models, we used stepwise elimination, where those predictors that contributed to the model at *p* < 0.2 were kept in the analysis. The first multivariable model included maternal anxiety and/or depression on at least one occasion (from pregnancy to child age 5 years), socio-economic factors (cohabitation status and parental education), and sex. In the following multivariable model, we added current episodes of maternal depression or anxiety, maternal ADHD symptoms, and child ADHD at 8 years. We also checked whether including child symptoms of anxiety and ADHD at 3.5 years, or interactions terms (between maternal anxiety and/or depression and maternal ADHD symptoms, and maternal anxiety and/or depression and child ADHD), altered the model. Odds ratios (ORs) and 95% confidence intervals (CIs) were computed. Tests were two-tailed, and significance levels were set at *p* < 0.05. Statistical analyses were performed using SPSS Statistics for Windows, version 26.

## Results

Of the 781 participating children, 91 (11.7%) were classified with at least one anxiety disorder at 8 years of age (Table 1). Within this group with anxiety, 33 (36.3%) were also classified with ADHD.

**Table 1:** Child psychiatric categories by CSI-4^a^ at 8 years of age (*n* = 781)

	*n*	%	*n*, boys/girls	%, boys/girls
Diagnosis				
Any anxiety disorder	91	11.7	41/50	10.0/13.5
Social phobia	15	1.9	7/8	1.7/2.2
Separation anxiety	22	2.8	12/10	2.9/2.7
Generalized anxiety disorder	32	4.1	19/13	4.6/3.5
Specific phobia	57	7.3	26/31	6.3/8.4
ADHD	85	10.9	55/30	13.4/8.1*
More than one anxiety disorder	25	3.2	15/10	3.6/2.7
Any anxiety and ADHD	33	4.2	14/19	3.4/5.1

^*^p = 018; ADHD, attention-deficit/hyperactivity disorder

ªCSI-4, Child Symptom Inventory-4

Compared with the children not classified with at least one anxiety disorder at age 8 years, significantly more children within the group with anxiety had mothers scoring above the SCL threshold (≥ 2) for anxiety and/or depression at all six time points (from pregnancy to child age 5 years), as well as at age 8 years, and above the ASRS threshold (≥ 14) for ADHD (Table 2).

**Table 2:** Descriptives and group comparisons between children classified with and without anxiety disorders

	No anxiety disorder 8 years		Anxiety disorder 8 years			
	*n*	% (*n*)	*n*	% (*n*)	χ	*p*
Maternal SCL ≥ 2						
Week 15 in pregnancy	674	8.6 (58)	90	18.9 (17)	9.48	0.002
Week 30 in pregnancy	663	4.2 (28)	88	11.3 (10)	8.25	0.004
Child age 6 months	660	6.8 (45)	87	14.9 (13)	7.09	0.008
Child age 18 months	641	8.4 (54)	85	23.5 (20)	18.71	< 0.001
Child age 3 years	675	10.8 (73)	90	32.2 (29)	31.49	< 0.001
Child age 5 years	627	6.4 (40)	79	30.4 (24)	49.92	< 0.001
Child age 8 years	628	9.6 (60)	83	28.9 (24)	26.4	< 0.001
Maternal ASRS-6 ≥ 14	673	5.3 (36)	88	17.0 (15)	17.03	< 0.001
Covariates						
Sex (girls)	690	46.4 (320)	91	54.9 (50)	2.37	0.124
Cohabitation status^a^	636	14.9 (95)	85	22.4 (19)	3.29	0.070
Parental education^b^	673	16.8 (113)	90	35.6 (32)	18.16	< 0.001
Child ADHD 3.5 years	690	(113)	91	(27)	9.66	0.002
		M (SD)		M (SD)	*Z*	
Child anxiety symptom sum scores 3.5 years	690	0.96 (1.43)	91	2.03 (2.66)	–3.93	< 0.001

^a^Cohabitation status, not living together with father

^b^Parental education, mean education length ≤ 12 years; SCL, short versions of the Hopkins Symptom Checklist; ASRS-6, the Adult Self-Report Scale-6 for attention-deficit/hyperactivity disorder (ADHD); child ADHD and anxiety symptom sum scores at 3.5 years were computed from the Preschool Age Psychiatric Assessment; disorders at 8 years were classified by the Child Symptom Inventory-4; M, mean; SD, standard deviation

At all six time points, the mothers of the children in the group with anxiety had significantly higher mean SCL scores compared with the mothers of the children without anxiety disorders at age 8 years (Fig. 1). Supplemental Fig. 1 shows the overall trend that when maternal ADHD symptoms was present (ASRS ≥ 14), mothers had significantly higher mean SCL scores compared with mothers below the ADHD threshold both when their child was classified with an anxiety disorder or not at age 8 years. In univariable regression analyses, maternal anxiety and/or depression (SCL ≥ 2) on at least one occasion, current maternal anxiety and/or depression, maternal ADHD symptoms (ASRS ≥ 14), and parental education (≤ 12 years) all contributed significantly to child anxiety disorder, but sex and cohabitation status did not (Table 3). In multivariable analyses, both maternal anxiety and/or depression on at least one occasion and maternal ADHD symptoms remained significant predictors of child anxiety with more than a twofold increase in ORs (2.09 and 2.79, respectively); adding child ADHD did not alter these contributions substantially (Supplemental Table 1). When adding child anxiety symptoms and ADHD at age 3.5 years to the model, maternal anxiety and/or depression on at least one occasion and maternal ADHD symptoms remained significant predictors of child anxiety at 8 years (ORs = 1.23 (95% CI [1.10–3.27], *p* = 0.022) and 2.56 (95% CI [1.16–5.63], *p* = 020)), respectively. There were no significant effect modifications of the multivariable model when adding interaction terms of maternal anxiety and/or depression and maternal ADHD symptoms, or maternal anxiety and/or depression and child ADHD (statistics not shown).

**Table 3:** Logistic regression (univariable and multivariable); Association between child’s anxiety disorder at 8 years and parents’ symptoms of anxiety and depression, and ADHD

Predictors	Univariable analyses			Multivariable analyses^f^		
	B (SE)	OR (95% CI)	p	B (SE)	OR (95% CI)	*p*
*Maternal psychiatric symptoms (n)*						
Maternal anxiety/depression						
≥ 1 episode^a^ (215)	1.10 (0.23)	2.99 (1.92–4.67)	< .001	0.74 (0.28)	2.09 (1.22–3.58)	0.007
Current episode^b^ (84)	1.35 (0.28)	3.85 (2.24–6.64)	< .001	0.96 (0.31)	2.60 (1.42–4.77)	0.002
Maternal ADHD^c^ (51)	1.29 (0.33)	3.64 (1.90–6.96)	< .001	1.03 (0.39)	2.79 (1.31–5.93)	0.008
*Socio-economic factors (n)*						
Cohabitation status^d^ (114)	0.51 (0.28)	1.67 (0.96–2.90)	0.072			
Parental education^e^ (145)	1.01 (0.24)	2.73 (1.70–4.40)	< .001	0.87 (0.27)	2.38 (1.40–4.05)	< 0.001
*Child factors (n)*						
Sex (girl) (370)	0.34 (0.22)	1.41 (0.91–2.19)	.125	0.50 (0.25)	1.65 (1.01–2.70)	0.046
*Constant*				–3.03 (0.24)	0.05	< 0.001

^a^Mean sum score on short versions of the Hopkins Symptom Checklist ≥ 2 in at least one of the assessments at week 15 or 30 in pregnancy; or at child age 6 months, 18 months, 3 years, or 5 years

^b^Mean sum score on short versions of the Hopkins Symptom Checklist ≥ 2 at child age 8 years

^c^Adult Self-Report Scale sum score ≥ 14

^d^Cohabitation status, not living together with father

^e^Parental education, mean education length ≤ 12 years

^f^Included in the multivariable analysis were participants with full datasets (*n* = 688, including 82 children classified with at least one anxiety disorder at age 8 years); ADHD, attention-deficit/hyperactivity disorder; disorders at 8 years were classified by the Child Symptom Inventory-4

**Fig. 2:**
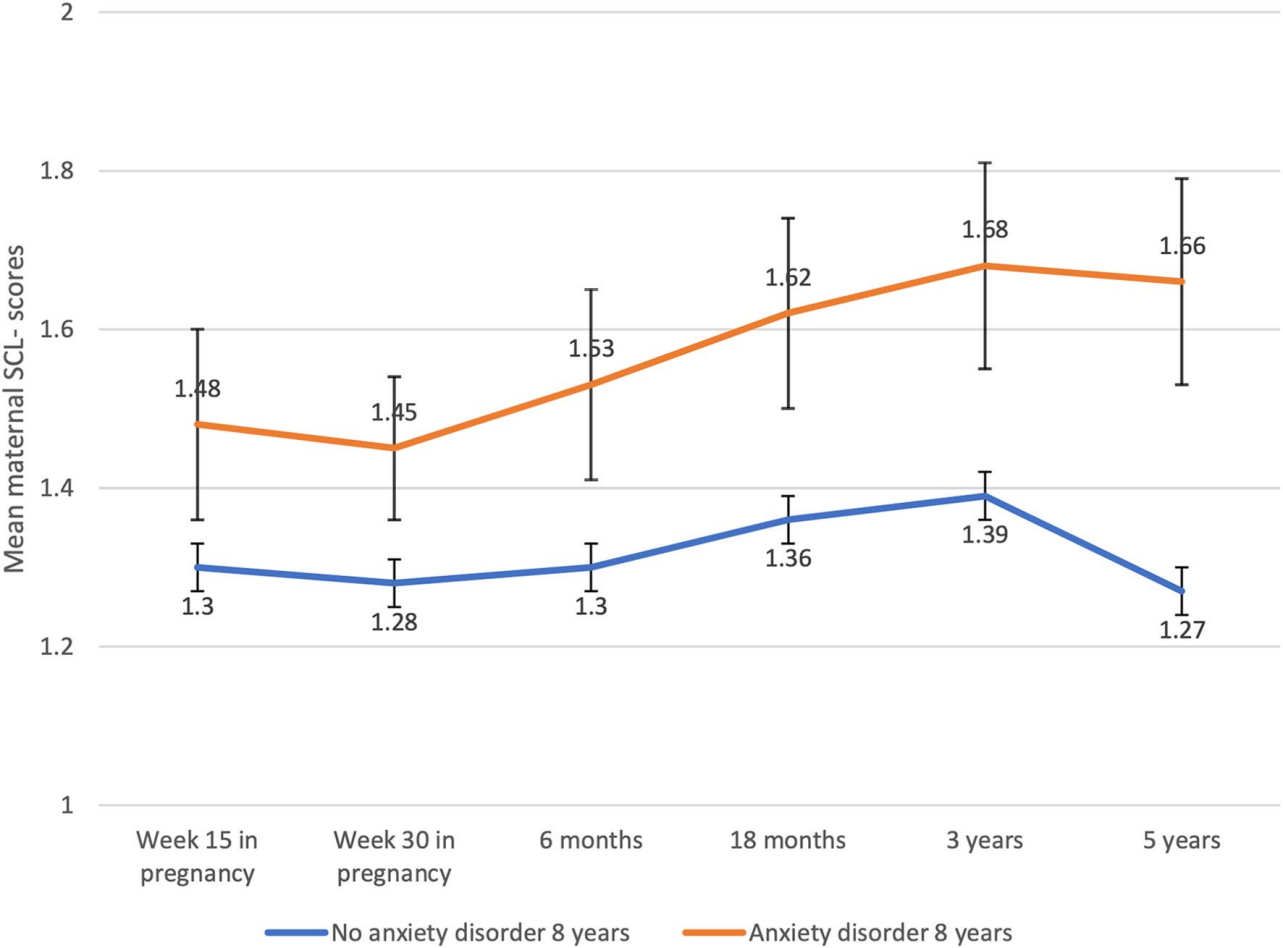
Anxiety or depressive symptoms (mean SCL scores with 95% confidence intervals) in mothers of children with and without anxiety disorder at age 8 years *SCL*, short versions of the Hopkins Symptom Checklist; disorders at 8 years were classified by the Child Symptom Inventory-4

Among the children, the proportion with an anxiety disorder at age 8 years increased incrementally with the number of episodes of anxiety and/or depression in mothers in a exposure–response manner (Fig. 2).

**Fig. 3:**
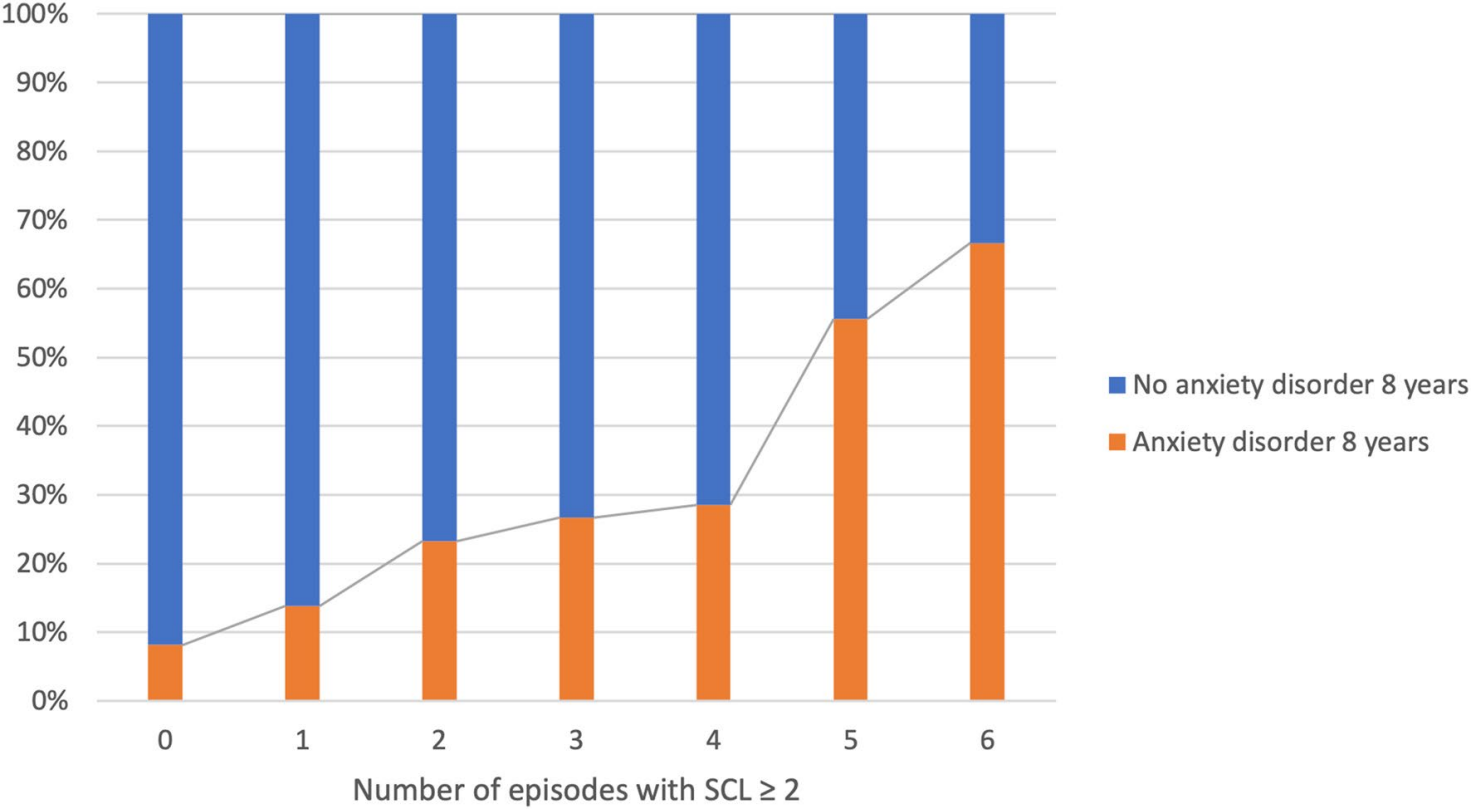
Proportion of children with anxiety at 8 years in mothers with and without repeated episodes of SCL ≥ 2 *SCL*, short versions of the Hopkins Symptom Checklist; disorders at 8 years were classified by the Child Symptom Inventory-4

Univariable logistic regression models gave increases in ORs for anxiety in the child for each maternal episode of anxiety and/or depression, but with larger and overlapping CIs (because of a gradual drop in the number of children for each maternal episode of anxiety and/or depression) (Supplemental Table 2). For example, the estimated OR for the association between exposure to three or more maternal episodes (*n* = 56) versus no episode and child anxiety at 8 years was 4.66 (CI [2.55–8.52], *p* = 0.001 (Fig. 3)

### Background results on non-participants

There were no significant group differences in the mean number of child symptoms of anxiety or ADHD at child age 3.5 years between participants and non-participants in the follow-up study to child age 8 years. Nor did we find significant differences related to child sex, parental cohabitation status, or the number of mothers with at least one episode of anxiety and/or depression between participants and non-participants, but there were slightly more parents with lower education in the non-participating group (25% vs. 19%, *p* = 0.02).

## Discussion

As hypothesized, we found that there was a significant association between maternal anxiety and depressive symptoms from pregnancy through early childhood and child anxiety disorders at child age 8 years. Considering the known heritability of anxiety disorders [32], this finding was not surprising and is consistent with a recent meta-analysis [7]. However, most studies discussed in that meta-analysis with internalizing symptoms as outcomes ended before 5 years, which the present study has expanded upon by including children at age 8 years.

We found that high anxiety and/or depression scores in mothers on at least one occasion from pregnancy to child age 5 years significantly predicted child anxiety at 8 years. Rogers et al. only found a significant association between postnatal maternal anxiety and depression and middle-childhood anxiety [7]. However, the importance of maternal symptoms later in early childhood was also supported by a Canadian study reporting a twofold increased risk of emotional disorders in adolescent offspring when exposed to maternal depression between the ages of 2–3 and 4–5 years [9]. A large French study (*n* = 1,183 mother–child dyads) using the brief Strengths and Difficulties Questionnaire found that depression in mothers during preschool age was related to child emotional problems at age 5 years [33]. Moreover, an Australian study found that maternal anxiety and depression during early childhood were associated with a small, but significant risk for the development of high anxiety–depression symptoms in offspring at age 14 years [1]. 

In the present study, the association between maternal anxiety and/or depression and offspring anxiety remained significant when maternal ADHD symptoms was included in the model. To our knowledge, maternal ADHD symptoms has not been controlled for in previous studies investigating this association, despite the high comorbidity rates between these disorders [34]. This comorbidity, together with the known genetic transmission of ADHD traits [35], suggests that it is important to include maternal ADHD symptoms when investigating the link between maternal anxiety and/or depression and anxiety of their offspring, the lack of which is a limitation of previous studies of risk factors [14, 36], as also noted by Robinson et al. in a recent review [15]. Indeed, we found that maternal ADHD symptoms significantly predicted child anxiety, even after controlling for maternal anxiety and/or depression and child ADHD, highlighting that maternal ADHD symptoms is a risk factor that warrants inclusion in studies of child anxiety.

Consistent with our hypothesis, we found that repeated episodes of maternal anxiety and/or depression increased the risk for an anxiety disorder in their offspring. Repeated exposure to maternal symptoms may increase the child risk through an intricate gene–environment interplay [32], although small group sizes at the different time points resulted in broad CIs suggesting caution in drawing firm conclusions. Nevertheless, this finding is consistent with a previous study that followed children up to the age of 14 years, suggesting that children exposed to persistent or repeated episodes of maternal anxiety and depression during early childhood were at increased risk of future anxiety [1]. Moreover, in their French study, van der Waerden et al. found that persistent depressive symptoms (either intermediate or high) in mothers were related to child emotional problems at age 5 years [33]. Taken together, these studies point to the maternal chronicity of depression as an important risk factor for child internalizing symptoms.

### Strengths and limitations

The strengths of the current study were its population-based cohort design and prospective follow-ups with multiple assessments of maternal symptoms to middle childhood, including common comorbidities in both mothers and children. To avoid overestimation of associations, we included both current maternal symptoms and child symptoms of anxiety and ADHD at 3 years. The present study also has several limitations. Selection bias due to attrition has been highlighted [17, 19]. However, we found no significant differences related to child sex, parental cohabitation status, or proportion of mothers with at least one episode of anxiety and/or depression between responders and non-responders when the children were 8 years old, but there were slightly more parents with lower education in the non-responding group (25% vs. 19%, *p* = 0.02). Although we examined maternal symptoms of anxiety and depression together, with high internal consistency, our scale could not discriminate between the two symptom clusters. Although the SCL-5 and SCL-8 are not diagnostic tools for anxiety and/or depression, the literature suggests that 50%–60% of individuals scoring above the cut-off on these instruments would most likely qualify for one or more mental disorders in clinical interviews [18, 37]. Mothers self-reported about ADHD symptoms only once (at child age 3 years). Even though the questionnaire specifically asks about these symptoms during the last 6 months, we were not able to consider possible fluctuations in maternal ADHD symptoms over longer periods. The participants in this study were oversampled for elevated symptoms of ADHD at 3.5 years, which might have excluded quiet or introverted preschoolers who were perhaps prone to anxiety, and this may have weakened our risk estimates. Children’s symptoms of anxiety and ADHD were reported by mothers only, adding the risk of shared method variance and reporter bias, as anxious or depressed mothers may report more symptoms in their children. However, child symptoms were assessed 3 years after the last maternal self-report, and in the multivariable logistic regression model, we controlled for current episodes of high maternal symptoms. Finally, the current study was not designed to detect causal associations, and we were not able to control for genetic transmission of anxiety symptoms between generations.

In conclusion, the findings of the present study link maternal anxiety, depression, and ADHD during pregnancy and early childhood to child anxiety at age 8 years. Treatment of maternal anxiety and depression, and prevention programs for their children, have been shown to benefit child mental health [38, 39]. The current study implies that paying attention to mothers with anxiety and/or depression, especially when recurring maternal symptoms are present, may also be important for the child several years later. Longitudinal studies are needed to determine which interventions in early childhood are effective.

## Supplementary Information

Below is the link to the electronic supplementary material.

Supplementary file1 (PDF 152 KB)

## Data Availability

The data that support the findings of this study were available from MoBa at the Norwegian Institute of Public Health, but restrictions apply to the availability of these data, used under license for the current study, and so are not publicly available.
