# Supplementary material for: Do maternal anxiety and depressive symptoms predict anxiety in children with and without ADHD at 8 years?
Source: Eur Child Adolesc Psychiatry. 2024 Feb 20;33(9):3169–78. doi: 10.1007/s00787-024-02374-1 (PMC11424742; doi:10.1007/s00787-024-02374-1)
Supplement: Supplementary file 1 — Supplementary file1 (PDF 152 KB) [file 787_2024_2374_MOESM1_ESM.pdf]

Supplementary Information: *Do maternal anxiety and depressive symptoms predict anxiety in 8-year-old children with and without ADHD?*
Submitted to European Child and Adolescent Psychiatry

Christine Baalsrud Ingeborgrud, M.D[1]; Beate Oerbeck, Ph.D; Svein Friis, M.D, Ph.D; Are Hugo Pripp, Ph.D; Pål Zeiner, M.D, Ph.D; Heidi Aase, Ph.D; Guido Biele, Ph.D; Søren Dalsgaard, M.D, Ph.D; Kristin Romvig Overgaard, M.D, Ph.D

[1]University of Oslo, Institute of Clinical Medicine, Oslo, Norway

Address correspondence to: Christine Baalsrud Ingeborgrud, University of Oslo, Institute of Clinical Medicine, Division of Mental Health and Addiction, Child and Adolescent Psychiatry Unit, Po box 1039 Blindern, 0315 Oslo, Norway
Telephone number: +47 40867208; Email: c.b.ingeborgrud@medisin.uio.no

Supplemental Figure 1. Mean maternal SCL scores with 95% confidence intervals; by maternal ADHD and/or child anxiety disorder.

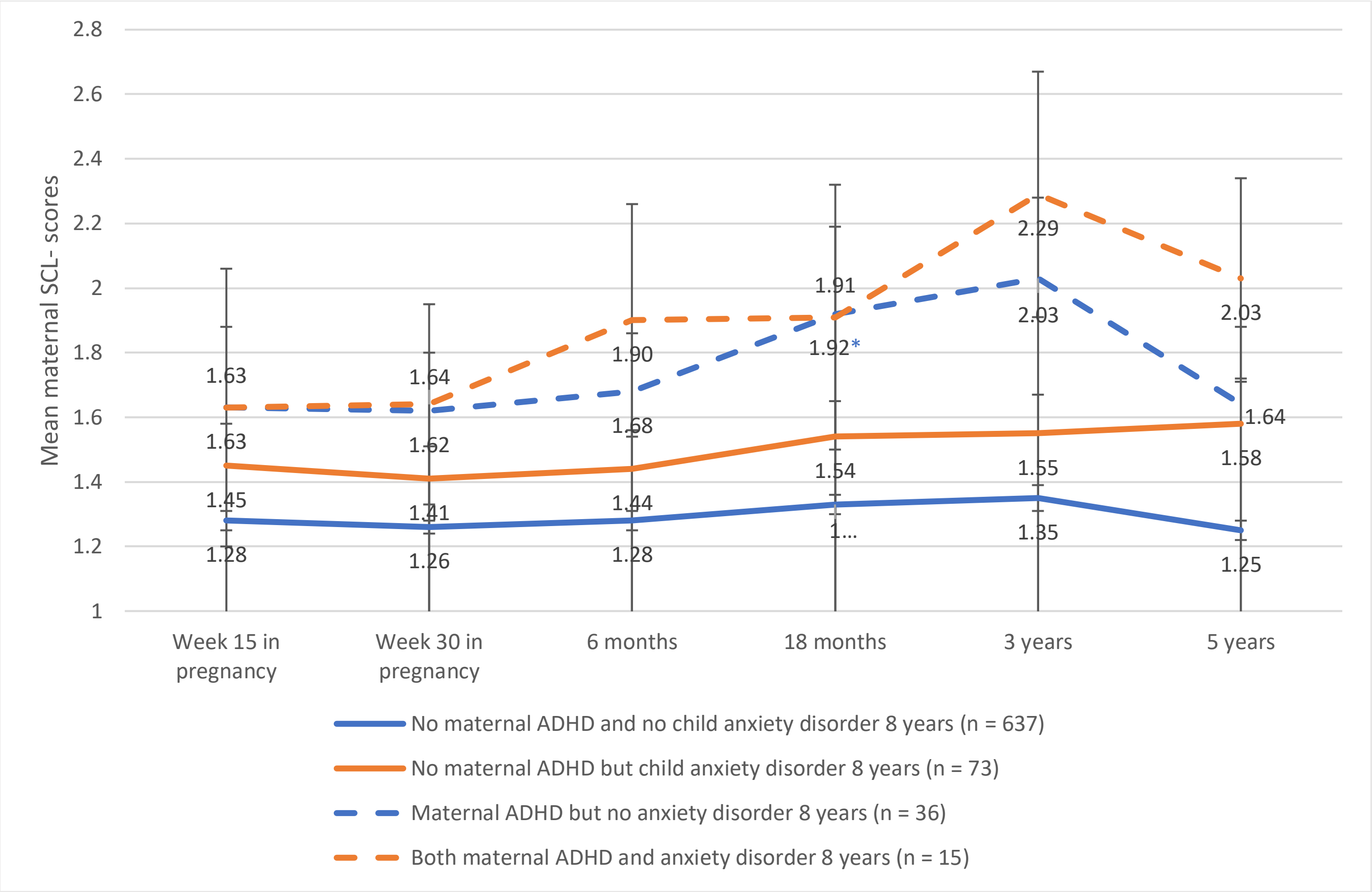

Note. SCL, short versions of the Hopkins Symptom Checklist; ADHD, attention-deficit/hyperactivity disorder; disorders at 8 years were classified by the Child Symptom Inventory-4.

Supplemental Table 1. Logistic regression (univariable and multivariable); Association between child's anxiety disorder at 8 years and parents' symptoms of anxiety and depression, and ADHD; child ADHD included in the multivariable analyses

Predictors	Univariable analyses			Multivariable analyses [g]		
	B (SE)	OR (95% CI)	*p*	B (SE)	OR (95% CI)	*p*
Maternal psychiatric symptoms (n)						
Maternal anxiety/depression						
≥1 episode [a] (215)	1.10 (0.23)	2.99 (1.92–4.67)	<.001	0.79 (0.29)	2.20 (1.24-3.90)	.007
Current episode [b] (84)	1.35 (0.28)	3.85 (2.24–6.64)	<.001	0.89 (0.33)	2.44 (1.28-4.64)	.007
Maternal ADHD [c] (51)	1.29 (0.33)	3.64 (1.90–6.96)	<.001	0.96 (0.41)	2.61 (1.16-5.86)	.021
Socio-economic factors (n)						
Cohabitation status [d] (114)	0.51 (0.28)	1.67 (0.96–2.90)	.072	-0.48 (0.36)	0.62 (0.31-1.25)	.181
Parental education [e] (145)	1.01 (0.24)	2.73 (1.70–4.40)	<.001	0.66 (0.30)	1.93 (1.08-3.44)	.026
Child factors (n)						
Sex (girl) (370)	0.34 (0.22)	1.41 (0.91–2.19)	.125	0.71 (0.27)	2.03 (1.20-3.43)	.008
Child ADHD 8 years (85) [f]	1.94 (0.26)	6.98 (4.18-11.65)	<.001	1.85 (0.32)	6.33 (3.86-11.84)	<.001
Constant				*-3.33 (0.26)*	*0.36*	<.001

Note. [a] Mean sum score on short versions of the Hopkins Symptom Checklist ≥2 in at least one of the assessments at week 15 or 30 in pregnancy; or at child age 6 months, 18 months, 3 years, or 5 years; [b] mean sum score on short versions of the Hopkins Symptom Checklist ≥2 at child age 8 years; [c] Adult Self-Report Scale sum score ≥14; [d] cohabitation status, not living together with father; [e] parental education, mean education length ≤12 years; [f] ADHD at 8 years was classified by the Child Symptom Inventory-4; [g] included in the multivariable analysis were participants with full datasets (n = 688, including 82 children classified with at least one anxiety disorder at age 8 years); ADHD, attention-deficit/hyperactivity disorder; disorders at 8 years were classified by the Child Symptom Inventory-4.

Supplemental Table 2. Univariable logistic regression: association between child anxiety disorder at age 8 years, and number of maternal episodes of anxiety and/or depression

Maternal anxiety/depression (n)	B (SE)	OR (95% CI)	*p*
0 episodes (566) [a]	–1.10 (0.23)	0.33 (0.21–0.52)	<.001
≥1 episode (215)	1.10 (0.23)	2.99 (1.92–4.67)	<.001
≥2 episodes (99)	1.42 (0.26)	4.14 (2.50–6.87)	<.001
≥3 episodes (56)	1.54 (0.31)	4.66 (2.55–8.52)	<.001
≥4 episodes (26)	1.82 (0.41)	6.19 (2.75–13.93)	<.001
≥5 episodes (12)	2.44 (0.60)	11.42 (3.54–36.77)	<.001
6 episodes (3)	2.74 (1.23)	15.48 (1.39–172.49)	<.001

Note. [a] Mean sum score on short versions of the Hopkins Symptom Checklist ≥2 in none of the assessments at week 15 or 30 in pregnancy; or at child age 6 months, 18 months, 3 years, or 5 years.
